# Challenging Diagnosis of Abdominal Actinomycosis: A Case Report of a 59-Year-Old Male With Acute Appendicitis-Like Presentation

**DOI:** 10.7759/cureus.70966

**Published:** 2024-10-06

**Authors:** Ahmet Erbagci, Ayşe Nur Toksöz Yıldırım

**Affiliations:** 1 Pathology, Istanbul Medeniyet University, Istanbul, TUR; 2 Pathology, Göztepe Research and Training Hospital, Istanbul, TUR

**Keywords:** abdominal actinomycosis, actinomyces, ileocecal band, misdiagnosis, pitfall

## Abstract

Abdominal actinomycosis, a rare and often misdiagnosed condition caused by *Actinomyces israelii*, typically a commensal organism in the oral cavity and gastrointestinal tract, can become pathogenic, leading to chronic granulomatous infections that mimic various abdominal pathologies, including malignancies. We present a case of a 59-year-old male with coronary artery disease and type 2 diabetes who presented with severe abdominal pain, initially diagnosed as acute appendicitis. During exploratory laparotomy, an ileocecal band mimicking a congenital anomaly was discovered. Histopathological examination confirmed abdominal actinomycosis, revealing clusters of *Actinomyces *bacteria surrounded by acute inflammatory cells. The patient was successfully treated with surgical intervention and prolonged penicillin therapy, with no recurrence during a four-month follow-up. This case highlights the diagnostic challenges posed by abdominal actinomycosis and emphasizes the importance of considering it in the differential diagnosis of abdominal masses and appendicitis-like symptoms.

## Introduction

*Actinomyces israelii*, a gram-positive bacillus of the *Actinomycetales *genus, was first identified in 1896 [1]. It is a normal commensal inhabitant of the human oral cavity, bronchial, and upper gastrointestinal tracts [2]. Despite its ubiquitous presence in these regions, it rarely causes disease and is seldom reported as a pathogen. However, when pathogenic, actinomyces can lead to the formation of fistulas and sinuses and may present as an abdominal mass or abscess.

In infected tissues, *Actinomyces *species form clusters of tangled filaments surrounded by polymorphonuclear neutrophils. The surrounding areas show subacute or chronic inflammation with granulation tissue and extensive fibrosis. Sulfur granules may be observed but are not exclusive to actinomycosis, as they can also appear in conditions like nocardiosis, botryomycosis, eumycetoma, and chromomycosis [3].

Abdominal actinomycosis most frequently presents as appendicitis [4]. It has also been reported to mimic colon malignancies [5]. Additionally, it has been observed in women with a history of intrauterine contraceptive device use, who are at increased risk [6]. This diagnostic challenge often necessitates surgical intervention, including resection, to confirm the nature of the mass.

In this case report, we present a 59-year-old male who demonstrated an acute appendicitis-like presentation caused by *Actinomyces*, which formed an ileocecal band mimicking acute appendicitis, a presentation distinct from those typically reported in the literature.

## Case presentation

A 59-year-old male patient with a medical history of coronary artery disease and type 2 diabetes presented to the emergency department with severe abdominal pain, which had begun the previous evening. The patient reported intermittent abdominal pain over the past six to seven months. On physical examination, there was tenderness in the right upper quadrant with a positive Murphy's sign. Laboratory tests showed a white blood count of 21 x 10^3^ /uL (normal range: 4-10 x 10^3^ /uL) and a neutrophil count of 18.53 x 10^3^/uL (normal range: 2-7 x 10^3^/uL). The patient was initially diagnosed with acute appendicitis and subsequently underwent an exploratory laparotomy.

During surgery, a band extending from the ileocecal region to the small intestines was observed, initially presumed to be a congenital band. The mesoappendix was attached to this band. A band excision and appendectomy were performed. Macroscopically, the appendix appeared edematous with a regular serosal structure. The band material was a soft, cream-yellow, heterogeneous tissue.

**Figure 1:**
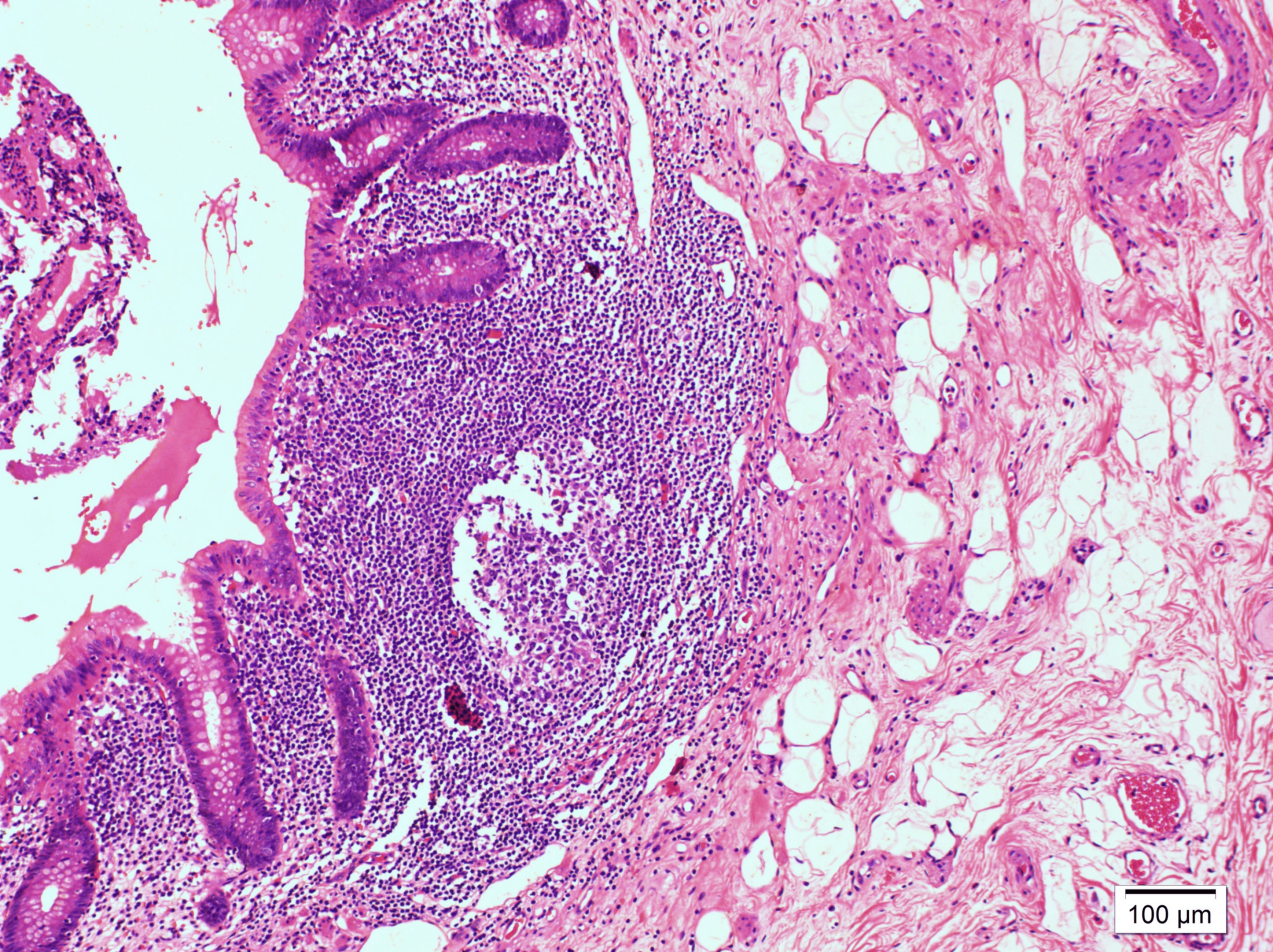
Histopathological examination of the appendix Hematoxylin and eosin stain at 100x magnification demonstrating reactive lymphoid changes without significant pathological alterations.

Histopathological examination of the appendix revealed reactive lymphoid hyperplasia without other pathological findings (Figure 1).

**Figure 2:**
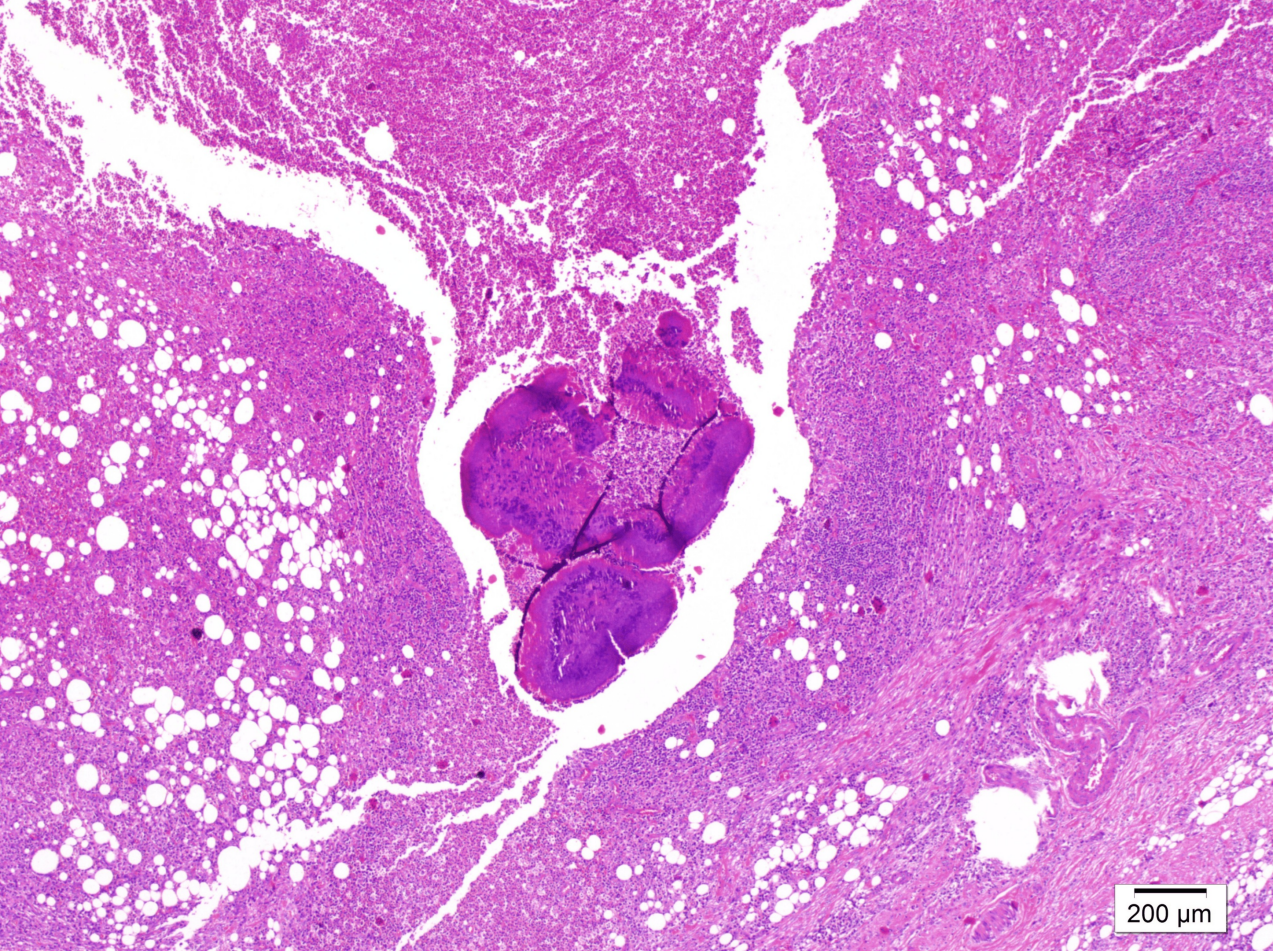
Fibrous tissue Hematoxylin and eosin stain at 40x magnification showing clusters of bacteria containing sulfur granules, surrounded by inflammatory cells, with artifactual clefts observed around the clusters.

However, the excised fibrous tissue, which was initially suspected to be a congenital band, contained rosette-like clusters of basophilic filamentous bacteria surrounded by abscessing acute inflammatory cells (Figure 2).

**Figure 3:**
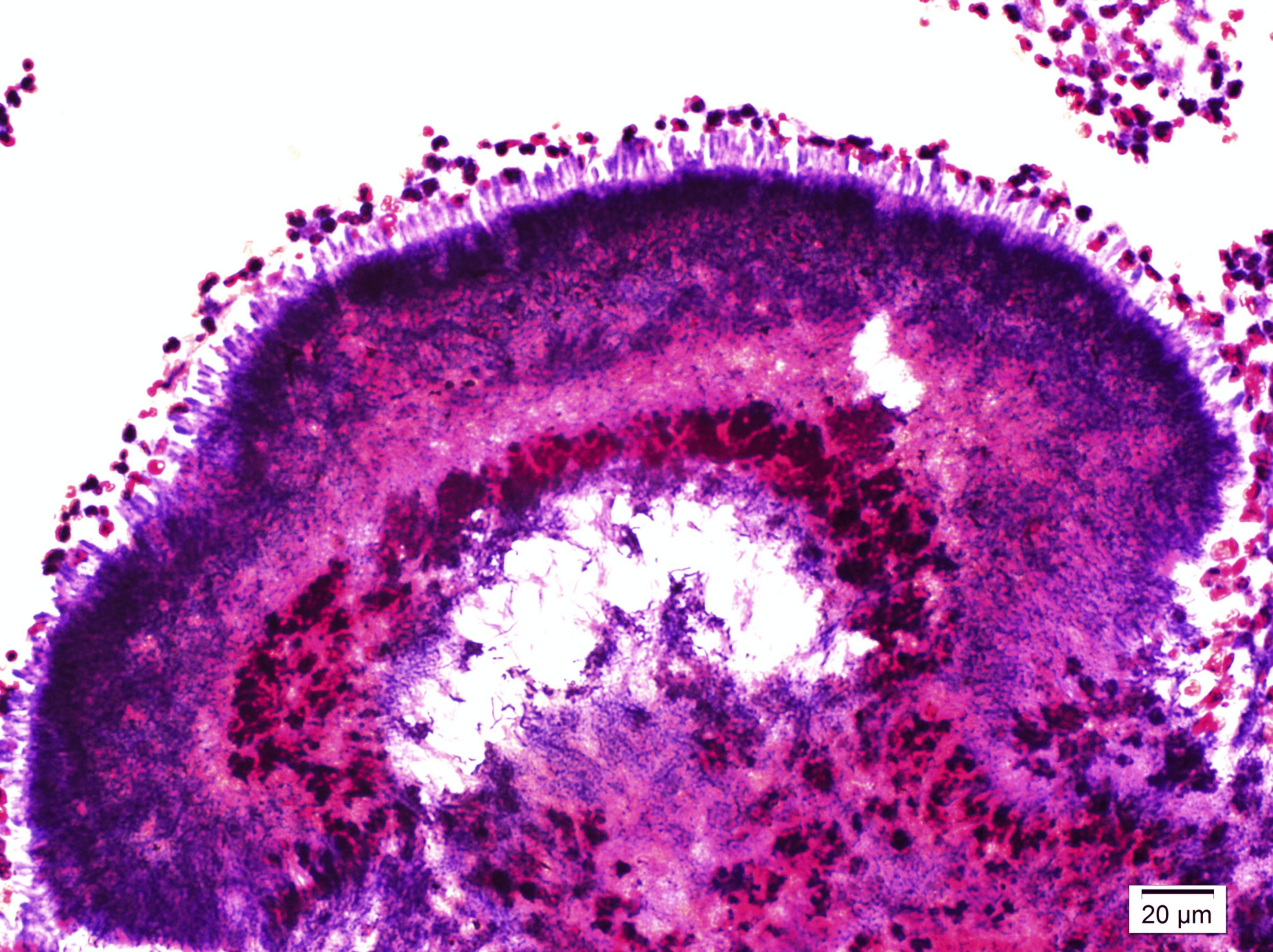
Gram stain The Gram-stained section at 200x magnification showing sulfur granules and filamentous bacterial clusters.

**Figure 4:**
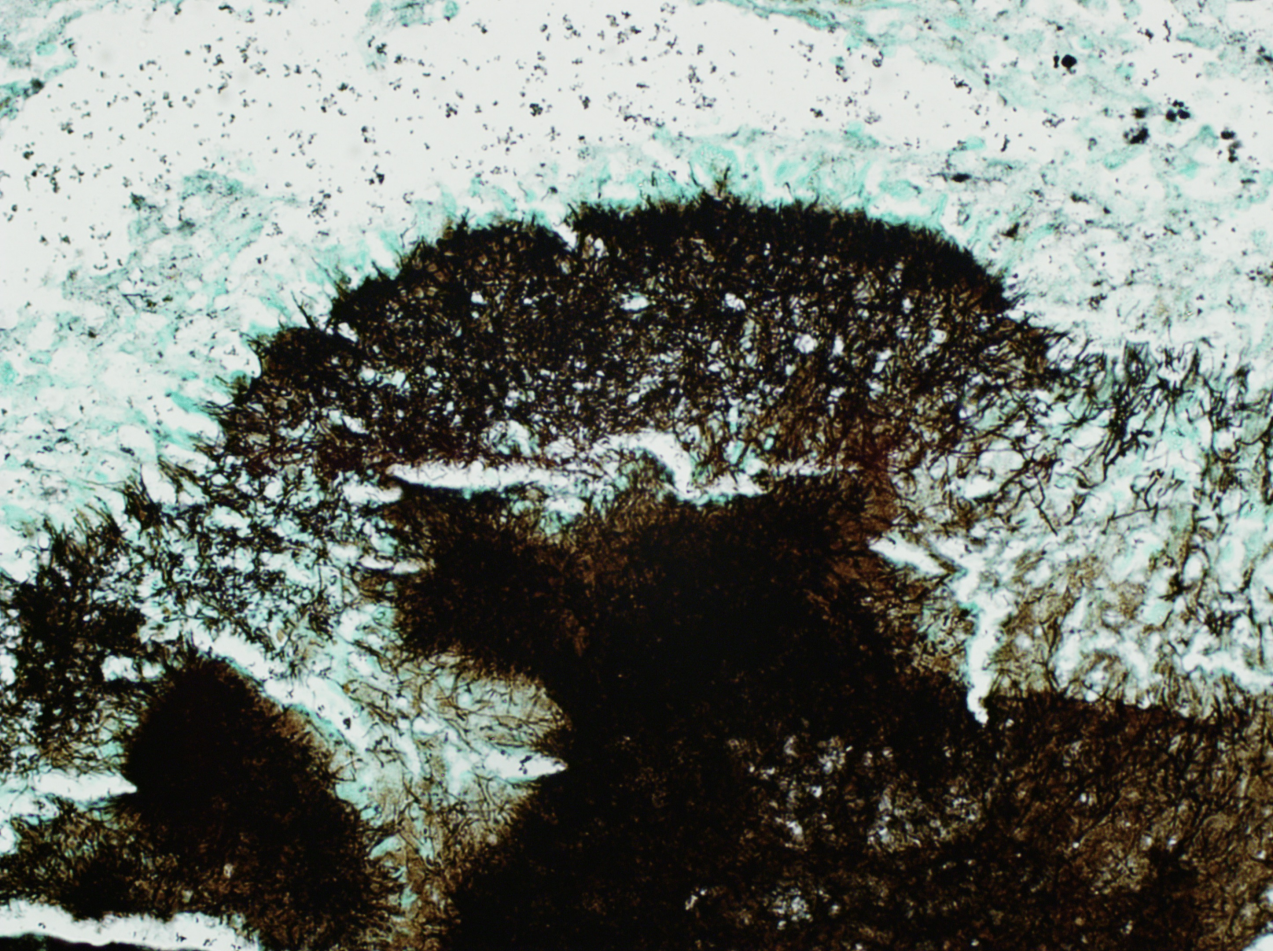
GMS stain The GMS stain at 400x magnification showing positively stained filamentous bacterial clusters. GMS: Grocott-Gömöri's methenamine silver.

These bacterial clusters were confirmed by Gram (Figure 3) and Grocott-Gömöri's methenamine silver (GMS) stains (Figure 4).

Based on these findings, the patient was diagnosed with abdominal actinomycosis. The patient received intravenous therapy with penicillin G at a dose of 24 million units per day for four weeks, followed by a transition to oral amoxicillin at 750 mg three times a day, planned for 12 months. All laboratory tests were within normal limits, and no signs of recurrence were observed during the four-month follow-up period.

## Discussion

Abdominal actinomycosis, caused predominantly by *Actinomyces israelii*, is a rare and often misdiagnosed infectious condition. This bacterium is a commensal organism commonly found in the oral cavity and gastrointestinal tract, but it can become pathogenic following mucosal disruption, leading to chronic granulomatous infections. In the abdomen, actinomycosis typically presents with non-specific symptoms and can mimic malignancies or other inflammatory diseases, which complicates its diagnosis.

In this case, the patient presented with symptoms initially suggestive of acute appendicitis, a common presentation for abdominal actinomycosis. However, the intraoperative findings of an ileocecal band mimicking a congenital anomaly, combined with histopathological evidence of actinomycosis, underscore the diagnostic challenge posed by this condition. The reactive lymphoid hyperplasia observed in the appendix is consistent with an inflammatory response, but the identification of basophilic filamentous bacteria in the excised tissue confirmed the diagnosis.

Preoperative diagnosis of abdominal actinomycosis remains challenging due to its rarity and the non-specific nature of its clinical presentation. Imaging studies may not always distinguish actinomycosis from malignancies or other pathologies. A contrast CT scan may sometimes reveal a solid mass, located either inside or outside the lumen, with regions of reduced density infiltrating adjacent tissues [7,8]. The radiological appearance often mimics that of malignancies, abdominal tuberculosis, or inflammatory bowel disease. The most typical findings on CT or barium studies include wall invasion leading to stricture formation, narrowing of the lumen caused by mass effect, and thickened mucosal folds. However, these features are also present in the previously mentioned conditions, making them not specific to actinomycosis [9].

The treatment of choice for actinomycosis is prolonged antibiotic therapy, typically with penicillin, to which *Actinomyces *species are highly sensitive. Penicillin is known to reduce morbidity and help prevent unnecessary invasive procedures, like laparotomy [10]. Nevertheless, in most cases, the diagnosis is made after surgery, yet it remains essential to complete the full course of penicillin [11]. Surgical intervention is often required to remove affected tissues, particularly when there is significant abscess formation or when the disease mimics a malignant process. In this case, the patient underwent appendectomy and excision of the involved tissue, followed by appropriate antibiotic therapy, which is expected to lead to a favorable outcome.

## Conclusions

This case underscores the diagnostic challenges posed by abdominal actinomycosis, particularly when it mimics more common conditions such as acute appendicitis. Preoperative diagnosis remains rare, and most cases are confirmed only after histopathological examination of surgically excised tissue. Imaging can be helpful, but it often fails to differentiate actinomycosis from other abdominal pathologies. Early recognition and a combination of surgical and prolonged antibiotic therapy are essential for favorable outcomes, as demonstrated in this case. Given its rarity and diagnostic difficulties, abdominal actinomycosis should always be considered in patients with atypical abdominal masses or recurrent inflammatory symptoms.

## References

[1] Israël J (1878). Neue beobachtungen auf dem gebiete der mykosen des menschen. Archiv F Pathol Anat.

[2] Schaal KP, Lee HJ (1992). Actinomycete infections in humans--a review. Gene.

[3] Smego RA, Foglia G (1998). Actinomycosis. Clin Infect Dis.

[4] Completo S, Veríssimo M, Pereira AMG, França I, Lemos PS (2022). Appendicular actinomycosis: behind the curtains of appendicitis. Cureus.

[5] Pamathy G, Jayarajah U, Gamlaksha DS, Constantine R, Banagala AS (2021). Abdominal actinomycosis mimicking a transverse colon malignancy: a case report and review of the literature. J Med Case Rep.

[6] Garner JP, Macdonald M, Kumar PK (2007). Abdominal actinomycosis. Int J Surg.

[7] Lee IJ, Ha HK, Park CM (2001). Abdominopelvic actinomycosis involving the gastrointestinal tract: CT features. Radiology.

[8] Ha HK, Lee HJ, Kim H, Ro HJ, Park YH, Cha SJ, Shinn KS (1993). Abdominal actinomycosis: CT findings in 10 patients. AJR Am J Roentgenol.

[9] Filippou D, Psimitis I, Zizi D, Rizos S (2005). A rare case of ascending colon actinomycosis mimicking cancer. BMC Gastroenterol.

[10] Atad J, Hallak M, Sharon A, Kitzes R, Kelner Y, Abramovici H (1999). Pelvic actinomycosis. Is long-term antibiotic therapy necessary?. J Reprod Med.

[11] Cintron JR, Del Pino A, Duarte B, Wood D (1996). Abdominal actinomycosis. Dis Colon Rectum.

